# The Red Blood Cell—Inflammation Vicious Circle in Sickle Cell Disease

**DOI:** 10.3389/fimmu.2020.00454

**Published:** 2020-03-13

**Authors:** Elie Nader, Marc Romana, Philippe Connes

**Affiliations:** ^1^Laboratoire Interuniversitaire de Biologie de la Motricité (LIBM) EA7424, Team Vascular Biology and Red Blood Cell, Université Claude Bernard Lyon 1, Université de Lyon, Lyon, France; ^2^Laboratoire d'Excellence du Globule Rouge (Labex GR-Ex), PRES Sorbonne, Paris, France; ^3^Université des Antilles, UMR_S1134, BIGR, Pointe-à-Pitre, France; ^4^Université de Paris, UMR_S1134, BIGR, INSERM, Paris, France

**Keywords:** sickle cell disease, inflammation, red blood cell, oxidative stress, heme

## Abstract

Sickle cell disease (SCD) is a genetic disease caused by a single mutation in the β-globin gene, leading to the production of an abnormal hemoglobin called hemoglobin S (HbS), which polymerizes under deoxygenation, and induces the sickling of red blood cells (RBCs). Sickled RBCs are very fragile and rigid, and patients consequently become anemic and develop frequent and recurrent vaso-occlusive crises. However, it is now evident that SCD is not only a RBC rheological disease. Accumulating evidence shows that SCD is also characterized by the presence of chronic inflammation and oxidative stress, participating in the development of chronic vasculopathy and several chronic complications. The accumulation of hemoglobin and heme in the plasma, as a consequence of enhanced intravascular hemolysis, decreases nitric oxide bioavailability and enhances the production of reactive oxygen species (ROS). Heme and hemoglobin also represent erythrocytic danger-associated molecular pattern molecules (eDAMPs), which may activate endothelial inflammation through TLR-4 signaling and promote the development of complications, such as acute chest syndrome. It is also suspected that heme may activate the innate immune complement system and stimulate neutrophils to release neutrophil extracellular traps. A large amount of microparticles (MPs) from various cellular origins (platelets, RBCs, white blood cells, endothelial cells) is also released into the plasma of SCD patients and participate in the inflammation and oxidative stress in SCD. In turn, this pro-inflammatory and oxidative stress environment further alters the RBC properties. Increased pro-inflammatory cytokine concentrations promote the activation of RBC NADPH oxidase and, thus, raise the production of intra-erythrocyte ROS. Such enhanced oxidative stress causes deleterious damage to the RBC membrane and further alters the deformability of the cells, modifying their aggregation properties. These RBC rheological alterations have been shown to be associated to specific SCD complications, such as leg ulcers, priapism, and glomerulopathy. Moreover, RBCs positive for the Duffy antigen receptor for chemokines may be very sensitive to various inflammatory molecules that promote RBC dehydration and increase RBC adhesiveness to the vascular wall. In summary, SCD is characterized by a vicious circle between abnormal RBC rheology and inflammation, which modulates the clinical severity of patients.

## Introduction

Sickle cell disease (SCD) is a genetic disease caused by a single mutation in the β-globin gene, leading to the production of an abnormal hemoglobin called hemoglobin S (HbS). Under deoxygenation, the HbS polymerizes, which causes the sickling of red blood cells (RBCs). Sickled RBCs are very fragile and rigid. These abnormal features of sickle RBCs are believed to be responsible for chronic anemia and frequent and recurrent painful vaso-occlusive crises, respectively. However, although the molecular defects at the origin of the disease have been well-described, patients with SCD may exhibit various acute and/or chronic complications, which may affect several organs, such as the lungs, heart, kidney, brain, skin, bones, and eyes, for example. It is worth noting that this genetic disorder is associated with an extreme inter-individual variability of its clinical presentation (1). In addition, while it is easy to consider that rigid RBCs could obstruct the microcirculation and trigger the onset of vaso-occlusive like events, it has been demonstrated that the transit time of RBCs in deoxygenated vascular areas would be theoretically too short to allow RBCs to spend enough time to sickle (2, 3). This means that other biological mechanisms participate in the pathophysiological processes of the disease. Activation and increased adhesiveness of neutrophils, monocytes and platelets to the endothelium, mainly in post-capillary venules, may initiate vaso-occlusion (4–7). The resulting decreased blood flow induces a longer transit time of RBCs in vascular areas with poor oxygen content, hence promoting HbS polymerization and RBC sickling (6). The accumulation of rigid RBCs and adherent circulating cells into the microcirculation is responsible for vaso-occlusion (6). Mounting evidence shows that SCD is characterized by the presence of chronic inflammation and oxidative stress, participating in the development of chronic vasculopathy, endothelial dysfunction and several chronic complications. In addition, this pro-oxidative and pro-inflammatory environment further impairs the rheological properties of RBCs, hence further impacting the clinical severity of disease in patients.

## The Role of Hemolysis in Inflammation and Vascular Dysfunction

Although chronic anemia is fairly well-tolerated by SCD patients, the severity of anemia modulates their aerobic fitness and quality of life (8, 9). Moreover, levels of anemia, partly determined by the rate of intravascular hemolysis in SCD patients, influences their survival rate (9). In addition, intravascular hemolysis also plays a key role in the pathophysiology of SCD, independently of its effects on anemia. Patients with the highest rate of hemolysis are at risk of earlier mortality, compared to those with less pronounced hemolysis (10). This section will discuss the consequences of enhanced hemolysis on inflammation, oxidative stress and the vascular function in SCD (11).

### Hemolysis and Nitric Oxide (NO) Bioavailability

NO produced by the endothelial NO-synthase (eNOS) is a strong modulator of vascular physiology. Through its effects on the vascular smooth cells, NO plays a key role in vasodilation. Moreover, NO has been shown to downregulate the transcription of several endothelial adhesion molecules of both the CAM (ICAM-1, VCAM-1) and selectin (E- and P-selectin) families (12) and to inhibit platelet activation (13). Accumulating evidence strongly supports a key role of hemolysis in the decrease of NO bioactivity/bioavailability in SCD (14, 15). The accumulation of hemoglobin in the plasma affects the bioavailability of nitric oxide (NO). Cell-free hemoglobin destroys NO at a rate of 1,000-fold faster than hemoglobin encapsulated in the RBCs (16). Moreover, hemolysis leads to the release of the arginase contained in erythrocytes into the plasma. The free arginase hydrolyzes arginine, which is the precursor to NO, to ornithine and urea, thereby exacerbating the decrease in NO bioavailability (11). Indeed, any decrease in NO bioactivity/bioavailability would result in vascular dysfunction.

Blood flow responses to sodium nitroprusside (a NO donor) or to L-NMMA (a NO-synthase inhibitor) are abolished in patients with SCD (17). Flow-mediated dilation response using nitroglycerin (a NO donor) is impaired in patients with SCD, compared to a control group (18). Belhassen et al. (19) reported increased diameter in the brachial artery at baseline in SCD patients, compared to a control group, but the vessel was not able to further dilate in response to a flow-mediated dilation procedure. At the microcirculatory level, Moeckesch et al. (20) reported decreased hyperemic response to skin heating localized stress in children with SCD, compared to healthy children, suggesting impaired microcirculatory NO-driven vasodilation in the former population.

The decrease in NO bioavailability could also be responsible for the enhanced platelet activation (13) observed in SCD patients, as documented by increased expression of platelet activation markers, such as P-selectin, CD63, activated glycoprotein IIb/IIIa, plasma soluble factor-3 and factor-4, β-thromboglobulin, and platelet-derived soluble CD40 ligand (21, 22). Such abnormal platelet activation has been associated with thrombosis and pulmonary hypertension, a clinical manifestation of endothelial dysfunction, in SCD patients (23). Kato et al. (24) also reported positive associations between the level of plasma soluble adhesion molecules and the severity of pulmonary hypertension.

On the whole, these studies strongly support a key role of hemolysis on endothelial/vascular dysfunction through its effects on NO bioactivity/availability.

### Hemolysis, Oxidative Stress, and Inflammation

The accumulation of extracellular hemoglobin and heme in SCD, which cannot be fully neutralized by haptoglobin and hemopexin, respectively (14), is a major source of oxidative stress. Hemoglobin may react with hydrogen peroxide through the Fenton reaction to form hydroxyl free radical and methemoglobin. The rate of autooxidation of Hb is greatly enhanced when released into the plasma, where it is partially oxygenated, and more particularly when the Hb tetramer dissociates into dimers (25). In addition, the repeated episodes of ischemia-reperfusion, such as those that occur during vaso-occlusive crises, induce the release of plasma xanthine oxidase (XO) (26). The released XO can impair vascular function by binding to the luminal cells of the vessel. This oxidative milieu results in exacerbated NO scavenging via an oxygen free radical-dependent mechanism, and further affects the vascular system. Mockesch et al. (20) recently showed that the impairment of microvascular regulation in children with SCD was significantly associated with both nitrotyrosine and markers of systemic oxidative stress, confirming the important roles of oxidative stress and NO scavenging in the development of vascular dysfunction in SCD.

Excessive production of reactive oxygen species (ROS) leads to endothelial damage, through peroxidation of the lipid membrane and/or DNA fragmentation, potentially leading to cellular apoptosis (27, 28). In addition, ROS play a central role in promoting vascular inflammation and endothelial activation through the activation of redox-sensitive transcription factors in the endothelium, such as NF-κB (29). The increased expression of several vascular cell adhesion molecules, such as VCAM-1, ICAM-1, L-, P-, and E-selectins, may then facilitate the binding of sickle RBCs, platelets and white blood cells (WBCs) to endothelial cells, which would trigger the onset of vaso-occlusion (30–32). Marui et al. (30) demonstrated that the use of pyrrolidine dithiocarbamate (an antioxidant) on cultured endothelial cells (HUVEC) was able to decrease the expression of VCAM-1 induced by IL-1β. There is clearly an interplay between oxidative stress and inflammation, which participates in the pathogenesis of SCD (33). Additionally, Belcher et al. (34) demonstrated that the administration of dimethyl fumarate (a drug activating Nrf2 expression and increasing the transcription and expression of several genes involved in antioxidant defenses) for several days in sickle cell mice decreased the hepatic expression of TLR4, NF-κB activation, VCAM-1, ICAM-1 and E-selectin mRNA levels, and hepatic necrosis.

Seminal work from Wagener et al. (35) showed that *in vitro* incubation of endothelial cells with heme led to a rise in adhesion molecule expression. Furthermore, the same group (36) reported that injection of heme in mice increased vascular permeability, adhesion molecule expression and leucocyte extravasation. Another group reported that incubation of endothelial cells with hemin (i.e., heme oxidized in its ferric form) increased the production of IL-8 (37). Although most of these inflammatory effects could be partly driven by the resulting enhanced oxidative stress caused by heme accumulation, heme would also directly activate the immune innate system (38).

Ghosh et al. (39) showed that hemin administration in sickle mice enhanced intravascular hemolysis, which further increased the amount of extracellular hemin, caused lung injuries typical of acute chest syndrome and decreased their survival rate. However, TLR4 inhibition (by the use of TAK-242) and hemopexin replacement therapy, prior to hemin infusion, protected sickle mice from developing acute chest syndrome. Chimeric sickle cell mice, knocked out for TLR4, did not develop extensive lung injury and were able to survive after infusion of hemin. Belcher et al. (40) investigated the role of heme in SCD vaso-occlusion and showed that administration of heme to SCD mice caused increased endothelial P-selectin and vWF expression, enhanced leucocyte rolling and adhesion and blood flow stasis. When treated with TAK-242 (an inhibitor of TLR4), blood stasis, leucocyte rolling and adhesion were decreased in mice injected with heme.

Adisa et al. (41) reported an association between plasma free heme concentration and the incidence of vaso-occlusive crises, in children with SCD. More recently, Pitanga et al. (42) reported a 4-fold higher level of circulating IL-1β in SCD patients at steady state, compared to healthy individuals. The authors also observed higher mRNA expressions of NLRP3 and IL-1β in the peripheral blood mononuclear cells (PBMC) of SCD patients, suggesting the activation of the NLRP3 inflammasome. Subsequently, they showed that incubation of PBMC with sickle RBCs induced higher mRNA expression of the genes encoding IL-1β, leukotriene, TLR9, NLRP3, caspase 1, and IL-18 in the supernatant, as compared to PBMC that were incubated with healthy RBCs. The authors did not look for the RBC element/molecule that could trigger the activation of the inflammasome and one could suggest that RBCs may contain several molecules that can act as eDAMPs. Hemolysis-related products are now considered as important eDAMPs that could trigger inflammasome activation in the context of SCD and participate in the pathophysiology of several complications (15, 43). Collectively, these findings suggest that hemolysis-related products could play a major role in the pathophysiology of several complications in SCD, through their binding to TLR4 and the activation of NF-κB and NLRP3 pathways and the enhanced production of pro-inflammatory cytokines, such as IL1β and IL18 (15). Other potent eDAMPs that may be released by RBCs during hemolysis include heat shock proteins (Hsp), such as Hsp70, IL-33, and adenosine 5′ triphosphate (43).

### Hemolysis, Neutrophil Extracellular Traps (NETs), and Inflammation

Heme/hemin have also been shown to activate neutrophils (44) and promote the release of NETs in SCD (45). Schimmel et al. (46) reported higher nucleosome levels in SCD patients at steady state, compared to healthy individuals, with a further increase during crisis. In addition, the authors reported a correlation between the levels of nucleosomes and the length of hospital stay in patients developing acute chest syndrome. NETs are composed of decondensed chromatin fibers coated with antimicrobial granular and cytoplasmic proteins, such as myeloperoxidase (MPO), neutrophil elastase, and alpha defensin. These NETs are able to promote endothelial activation, thus increasing VCAM-1 and ICAM-1 expression (47). It has also been suggested that NETs could promote vessel occlusion by providing a scaffold for platelets, RBCs and pro-coagulant molecules (48). Recent studies also demonstrated that NETs induced the activation of the NLRP3 inflammasome in macrophages through TLR4/TLR9 signaling pathways, leading to higher production of IL-1β in the context of diabetes and atherosclerosis (49, 50). Indeed, one may suspect a role of NETs in SCD pathogenesis through an activation of the immune innate system (15).

### Hemolysis and the Alternative Complement Pathway

Hemolysis activates the alternative complement pathway. In atypical uremic hemolytic syndrome, heme has been shown to activate the complement system in plasma and on endothelial cells (51). Heme-induced exocytosis of Weibel-Palade bodies from endothelial cells induces the expression of P-selectin, which is known to bind C3b and trigger complement activation (51). In addition, heme can trigger the release of C5a and C5b9, leading to the activation and permeabilization of endothelial cells (51). The attachment of membrane attack complexes to the endothelial cells may promote inflammation through NF-κB signaling (52).

Increased soluble C5b-9 levels have been reported in SCD patients (53, 54). Vercellotti et al. (55) demonstrated increased C3 activation fragments and C5b-9 in the kidneys, lungs and liver of sickle cell mice, compared to control mice, and Lombardi et al. (56) found increased microvascular deposition of C5b-9 on skin biopsies in SCD patients. Increased alternative pathway Bb fragments have also been reported in the plasma of both sickle cell mice and sickle cell patients (55, 57). The infusion of recombinant C5a has been shown to cause blood stasis and inflammation in the liver of sickle cell mice (through NF-κB activation and increased expression of TLR4 and several adhesion molecules), but this response was reversed by an anti-C5a receptor IgG (55). The increased externalization of phosphatidylethanolamine and phosphatidylserine at the membrane of sickle RBCs is also suspected to induce complement activation with increased C3 and C3b binding (56, 57). A very recent work investigated the role of heme on complement activation in the context of SCD (58). The authors showed increased C3 and C9 deposition in the kidneys of both sickle cell mice and SCD patients and demonstrated that C3 fragment deposition was increased in the kidney of normal mice receiving phenylhydrazine to promote intravascular hemolysis. The effects of hemin were tested on endothelial cells and it was shown that heme triggered rapid P-selectin, C3aR and C5aR expression, C3 and C5b9 deposition, and downregulated CD46, a transmembrane protein able to bind and inactivate C3b and C4b. The use of hemopexin with hemin reduced the deposition of C3 and C5b9 on endothelial cells. Merle et al. (59) demonstrated that P-selectin drives complement attack on endothelial cells during intravascular hemolysis in a TLR-4/heme-dependent manner. Altogether, these studies support a key role for hemolysis in endothelial dysfunction in SCD with implications for the participation of the alternative complement pathway.

### Hemolysis, Microparticles, Inflammation, and Oxidative Stress

Circulating extra-cellular vesicles (EV), such as microparticles (MPs, 0.1–1 μm) and exosomes (30–100 nm), are thought to play a role in the pathogenesis of SCD (60, 61). Several groups reported a 3- to 4-fold increase of plasma MPs (mainly originating from platelets and RBCs) in SCD patients at steady-state compared to healthy individuals (62–66), with a further rise during vaso-occlusive crises (62, 67, 68). Khalyfa et al. (69) reported increased levels of circulating exosomes in SCD patients compared to healthy individuals, with the most severe patients (i.e., with the highest rate of painful vaso-occlusive crises) exhibiting the highest levels.

MPs and exosomes carry diverse cargoes including proteins, RNA species, such as mRNA and miRNA and lipids that can be transported and exchanged between cells, strongly suggesting that EV play key roles in cell-cell communication at both paracrine and systemic levels (61, 70). Not specific to SCD, these EV may promote inflammation, oxidative stress, coagulation, and endothelial activation. The high amount of externalized phosphatidylserine at the surface of most of the MPs is responsible for their pro-coagulant property while others express tissue factor (60, 61). Various blood cell-derived MPs have also been shown to regulate the production of reactive oxygen species and promote endothelial activation (61, 71). MPs shed by endothelial cells (71), monocytes (72), and lymphocytes (73) induce endothelial ${\text{O}}_{2}^{-}$ and H_2_O_2_ production in cultured endothelial cells through processes involving different enzymatic systems, and thus may lead to apoptosis (74). Treatment of endothelial cells with platelet- and endothelial cell-derived MPs were associated with increased expression of cell adhesion molecules and monocyte-endothelial cell interactions (74, 75).

However, only a few studies have investigated the effects of EV in the context of SCD, and more particularly the effects of RBC-derived MPs. It seems that the amount of circulating RBC-derived MPs is directly related to the degree of hemolysis (64, 76, 77). Several authors reported strong associations between various markers of hemolysis, such as heme, lactate dehydrogenase, plasma hemoglobin, serum bilirubin, reticulocyte count, fetal Hb or hemoglobin concentration, and RBC-MPs (76, 77). Camus et al. (78, 79) previously demonstrated that *ex-vivo* generated sickle RBC-MPs, when infused in sickle cell mice, promoted kidney vaso-occlusions. The authors further demonstrated that these RBC-MPs delivered toxic heme to endothelial cells, which increased the production of reactive oxygen species and the expression of endothelial cell adhesion molecules, and promoted apoptosis. Interestingly, heme-loaded MPs were also shown to activate the alternative and terminal complement pathway at the surface of the endothelial cells (58). Khalyfa et al. (69) demonstrated that exosomes isolated from SCA patients with frequent vaso-occlusive crises, for which RBC-derived exosomes being the most abundant, decreased endothelial permeability and promoted P-selectin expression on cultured endothelial cells. These exosomes also significantly increased the adhesion of monocytes to the vascular wall in mice, compared with exosomes isolated from SCA patients with a less severe phenotype.

Taken together, these findings suggest that the accumulation of RBC-MPs, consecutive to enhanced hemolysis, in SCD could cause serious damage to the vascular system and modulate clinical severity.

## How do Sickle RBCs React to This Pro-Inflammatory and Pro-Oxidative Environment?

Because RBCs are very fragile and prone to lysis, SCD patients are characterized by chronic anemia. We previously discussed the consequences of enhanced hemolysis in SCD on the reduction of NO bioavailability, the increase in oxidative stress and inflammation, the production of NETs, the activation of the alternative complement pathway and the release of RBC-derived MPs, which all lead to endothelial activation and vascular dysfunction. However, this pro-inflammatory and pro-oxidative environment may further damage the RBCs, which could further alter their rheological properties and increase their fragility.

### Nitric Oxide and RBCs

The effects of NO on the vascular system have been well-described in the literature, but NO may also affect the mechanical properties of RBCs. One of the first reports suggesting that NO could affect RBC deformability was the study of Starzyk et al. (80), which demonstrated that intravenous infusion of L-NAME (an eNOS inhibitor) in rats caused a reduction in RBC deformability. Bor-Kucukatay et al. (81) then demonstrated that several eNOS inhibitors decreased RBC deformability. A recent work conducted in SCD showed that *in vitro* incubation of RBCs with sodium nitroprusside (a NO donor) decreased the amount of intracellular reactive oxygen species and increased RBC deformability (82). This study also demonstrated that, in addition to its effects on HbF production and the reduction of HbS polymerization, the positive effects of hydroxycarbamide treatment on SCD RBC deformability could be related to the increased NO delivery from the drug to sickle RBCs.

It has been suggested that the effect of NO on RBC deformability could be partially mediated by soluble guanylyl cyclase (sGC) (83), but studies by Bor-Kucukatay et al. (81) and Baskurt et al. (84) also support a role for NO in potassium permeability. In addition, NO could decrease the risk for hemolysis and increase RBC survival rate through its effects on eryptosis since NO is able to down-regulate caspase 3 activity through S-nitrosylation (85). More recently, another group demonstrated that the NO donor, sodium nitroprusside, inhibited the decrease in RBC deformability induced by ionophore A23187-mediated calcium influx in RBC (86). Increased intracellular calcium concentration activates calcium-sensitive K^+^ (Gárdos) channels, resulting in potassium-efflux and decreased cell volume, which in turn increases the stiffness of RBC; however, the presence of sodium nitroprusside abolished this calcium-induced impairment in RBC deformability (86). Barodka et al. (86) suggested that sodium nitroprusside may have limited calcium influx, thereby inhibiting the activation of Gárdos channels, and thus, maintaining cell volume and RBC deformability. However, the effects of NO on RBC rheology may not be limited only to its effects on RBC deformability. For instance, Bor-Kucukatay et al. (87) demonstrated that incubation of RBC with sodium nitroprusside decreased RBC aggregation, while giving L-NAME to rats resulted in a rise in their RBC aggregation. The underlying mechanisms at the origin of these findings are unclear, but might involve membrane/cytoskeletal protein nitrosylation or oxidative stress modulation. In conclusion, the reduction of NO bioavailability in SCD probably plays a role in the modulation of RBC rheology (82, 88).

### Oxidative Stress and RBCs

As previously discussed, oxidative stress is increased in SCD, both in plasma and RBC (15, 82, 89–92), with a further rise during painful vaso-occlusive crises (68). Through its effects on the membrane of RBCs (i.e., lipid peroxidation and protein oxidation) and caspase 3 activation (93, 94), oxidative stress is a key modulator of RBC rheological properties. Moreover, oxidative stress is able to activate Ca^2+^-permeable non-selective cation channels in the RBC membrane, leading to the accumulation of Ca^2+^ within RBCs, which can trigger RBC membrane scrambling, resulting in phosphatidylserine exposure and possibly in membrane bubbling and emission of MPs (95). In addition, the activation of Ca^2+^-sensitive K^+^ channels can lead to K^+^ exit, hyperpolarization, Cl^−^ exit and cell shrinkage (95).

Baskurt et al. (96) demonstrated that superoxide anion caused a decrease in RBC deformability, a slight decrease in RBC aggregation and a large increase in RBC aggregates strength, meaning that the RBC aggregates formed are more robust upon oxidative stress. Depending on the concentration used, hydrogen peroxide may decrease RBC deformability (high concentration) or increase RBC adhesion to endothelial cells (low concentration) (97). Using atomic force microscopy, Sinha et al. (98) demonstrated the deleterious effects of several oxidant molecules (hydrogen peroxide, diamide, primaquine bisphophate, and cumene hydroperoxide) on RBC cytoskeletal architecture and membrane stiffness. All these changes may affect the fragility of RBCs. For instance, McNamee et al. (99) recently showed that phenazine methosulfate (an agent that generates superoxide anion within RBCs) decreased RBC deformability and increased the sensitivity of RBCs to shear-mediated damage. Hierso et al. (100) compared the biophysical response of healthy and SCD RBCs to *in-vitro* oxidative stress, using t-butyl hydroperoxide (TBHP). TBHP increased the production of ROS and decreased GSH content within the RBCs of both SCD and healthy individuals. In addition, the molecule decreased RBC deformability and RBC aggregation, and increased the strength of RBC aggregates in the two populations. However, the magnitude of changes in RBC rheology was 2- to 3-fold higher in SCD patients than in healthy individuals, indicating that RBC from SCD patients are more susceptible to oxidative stress than RBC from healthy individuals. The decrease in RBC antioxidant defenses in SCD could account for these differences (100).

### Inflammation and RBCs

SCD is characterized by a pro-inflammatory state leading to high plasma cytokines levels. Karsten et al. (101) recently showed that 46 cytokines can be detected in RBCs lysates of healthy individuals, and their median concentrations in RBCs were 12-fold higher than in plasma. Among them, the authors reported the presence of IFN-γ, IL-1β, IL-18, TNF-α as well as several chemokines, such as IL-8 and RANTES. The mean IL-1β and IL-18 concentrations in whole blood were 0.5 and 13.3 pg/ml, respectively, but the concentrations reached 4.2 and 657.6 pg/ml in RBCs, respectively (after correction for white blood cell contamination). When incubated in a protein-free media, the authors demonstrated that RBCs were able to release TNF-α, RANTES, IL-6, IL-8, and other molecules. It was also demonstrated that RBCs were able to capture various recombinant cytokines by using a recombinant standard cytokine mix. This study concluded that RBCs are dynamic reservoirs of cytokines, preventing chemokine clearance and thereby prolonging chemokine half-life in the blood. One major locus for cytokines binding is the Duffy Antigen receptor for chemokines (DARC) (102). Instead of acting as a reservoir, Darbonne et al. (102) proposed that RBCs act as a sink for IL-8, thereby inactivating the IL-8-dependent gradient and preventing neutrophil recruitment. DARC may also capture other chemokines of the CXC and CC families (103). Lee et al. demonstrated that patients lacking erythroid DARC expression exhibited higher plasma chemokine levels following LPS exposure, suggesting that DARC could act as chemokine scavengers to decrease immune-activating signals (sink hypothesis). The two models for RBC regulation of cytokines and chemokines levels could appear contradictory, but they may be not mutually exclusive. Fukuma et al. (104) proposed that RBCs would scavenge chemokines/cytokines from sites of inflammation, but could eventually release them in response to a reduction of plasma levels, effectively maintaining homeostasis. The degree of rupture of this homeostasis is unknown in SCD, but some studies have investigated the consequences of various inflammatory molecules on RBC properties. Bester et al. (105, 106) recently demonstrated that IL-8 affects the shape of healthy RBCs with morphological changes typical of those occurring during eryptosis. Circulating extracellular histones (i.e., a marker of NETosis) have recently been reported to promote eryptosis in healthy donors, ending with increased RBC phosphatidylserine externalization and RBC shrinkage (107). Test et al. (108) demonstrated increased binding of C5-b7 and of C9 to dense sickle RBCs, increasing the susceptibility of these cells to C5b-9-mediated reactive lysis initiated by C5b6. George et al. (109) tested the effects of transforming growth factor β1 and endothelin-1 (two cytokines known to be elevated in the context of SCD) on healthy RBCs. They demonstrated that these two inflammatory molecules stimulated RBC NADPH oxidase activity, leading to the accumulation of reactive oxygen species, which are known to damage the membrane of RBCs and increase their rigidity when produced in excess. In addition, endothelin-1 has been shown to promote dehydration of sickle RBCs through an activation of the Gárdos Channel, leading to a rise in RBC density (110). Durpes et al. (111) showed that the percentage of RBCs with densities higher than 1.12 (i.e., irreversibly sickle dehydrated RBCs) was 17-fold higher in sickle cell patients expressing DARC, compared to Duffy-negative patients. Since chemokines and cytokines would be able to bind to DARC, these results suggest a link between inflammation and sickle RBC dehydration. Furthermore, the authors demonstrated that both IL-8 and RANTES promoted dehydration in sickle RBC expressing DARC, through an activation of the Gárdos pathway. The same group (112) reported an effect of these two chemokines on the activation of α4β1 integrin in sickle reticulocytes expressing DARC, resulting in a greater adhesion of sickle RBCs to immobilized VCAM-1 and fibronectin. These findings could partly explain why Drasar et al. (113) reported that SCD patients with RBCs expressing DARC could be more prone to developing leg ulcers and kidney disease than Duffy-negative SCD patients, although this association between Duffy phenotype and SCD clinical severity is still debated (114, 115). Nebor et al. (116) failed to find an association between Duffy phenotype and the clinical severity in a large cohort of SCD patients, but they reported higher plasma IL-8 and RANTES levels in Duffy-positive vs. Duffy-negative patients, suggesting that RBCs can clearly modulate the level of inflammation in SCD. Although the exact mechanisms by which RBCs can modulate inflammation in SCD are not fully understood, these findings support the fact that pro-inflammatory molecules may promote sickle RBC dehydration and increase RBC density/rigidity through increased Gárdos channel activity, as well as increase RBC adhesiveness to endothelial cells through α4β1 clustering.

## Impaired RBC Rheology Is Involved in the Pathophysiology of SCD

As previously discussed, enhanced hemolysis, due to the decreased deformability and increased fragility of sickle RBC, disturbs NO metabolism and promotes oxidative stress and inflammation through various mechanisms. In turn, this pro-inflammatory and pro-oxidative environment may further impair the rheological properties of RBCs and increase their fragility, further impacting on the clinical expression of the disease. For instance, SCD patients with the lowest RBC deformability have been reported to be at higher risk of developing priapism, leg ulcers and glomerulopathy than those with the highest RBC deformability (117–119). There is a clear relationship between RBC deformability, RBC fragility and the extent of hemolysis in SCD (120). Patients with the lowest deformability have higher hemolytic rates, which may increase their risk of developing hemolytic-like complications, such as those cited above (14, 121, 122). Moreover, abnormal RBC aggregation properties may also play a role in SCD pathogenesis and are modulated by both oxidative stress and inflammation. Lamarre et al. (123) found an association between increased RBC aggregation strength and the occurrence of acute chest syndrome. More recently, Lapouméroulie et al. (124) observed a rise of RBC aggregation and of the robustness of RBC aggregates in SCD patients during vaso-occlusive crisis compared to the steady-state condition. Abnormal RBC aggregation may both disturb blood flow in the microcirculation and microcirculation (125). In the microcirculation, increased RBC aggregate strength may increase vascular resistance and decrease blood flow at the entry of capillaries (88). In the macrocirculation, the different hemodynamic re-arrangements between flowing RBC aggregates and single flowing RBCs create a situation where the width of the cell free layer close to the vascular wall is larger when RBC aggregates are flowing, leading to a decrease in wall shear stress, and reductions in eNOS activation and NO production, thus impacting on the ability of the vessels to adapt their diameters (125–127).

## Conclusion

**Figure 1:**
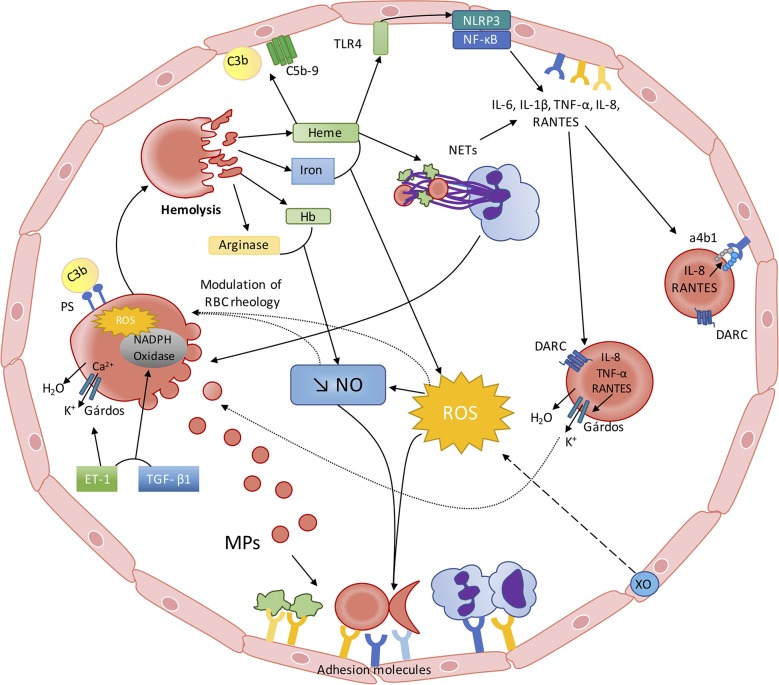
The red blood cell—inflammation vicious circle in sickle cell disease. *From RBC alterations to oxidative stress, inflammation and endothelial dysfunction*: Sickled RBC are very fragile and prone to hemolyze. Hemolysis leads to the release of heme, iron, Hb and arginase into the plasma, which interfere with the metabolism/bioavailability of NO: (I) free arginase may hydrolyze the NO precursor Arginine; (II) free Hb scavenges NO at a rate of 1,000-fold faster than Hb encapsulated in the RBCs; (III) heme and iron increase ROS generation, which lead to the production of peroxynitrite. ROS production is also enhanced by Xanthine Oxidase activation, caused by the repetition of ischemic/reperfusion events. Decreased NO bioavailability and increased ROS activate endothelial cells, which in turn express adhesion molecules of both the CAM and Selectin families, promoting cell-cell interactions. Free heme is able to activate endothelial TLR4, which promotes inflammasome activation and cytokines production through NF-κB activation. Heme may also activate neutrophils, which would release NETs that can also affect endothelial cells and act as a scaffold for platelets and RBCs. Recent evidence also showed that free heme could stimulate the complement pathway with potential consequences at the endothelial cell level. *From inflammation and oxidative stress to RBC alterations*: This pro-inflammatory and pro-oxidative environment, resulting from sickle RBCs alterations, also impacts on RBC rheology and physiology. Increased ROS production may lower RBC deformability and increase RBC aggregation. Decreased NO bioavailability could also participate in the decrease of RBC deformability and promote eryptosis. NETs could also promote RBC eryptosis. Circulating inflammatory molecules, such as ET-1 and TGF-β, may activate RBC NADPH Oxidase, which in turn would produce ROS and further alter RBC. ROS and ET-1 are known to activate the RBC Gárdos channel, which could favor RBC dehydration and further promote HbS polymerization. The enhanced release of MP by sickled RBCs could further exacerbate inflammation and oxidative stress. Increased RBC phosphatidylserine exposure may favor the binding of complement proteins at the surface of RBCs, which can induce their lysis. RBCs also act as a reservoir and/or a sink for pro-inflammatory cytokines/chemokines. IL-8, TNF-α, and RANTES promote RBC dehydration through Gárdos channel activation in RBCs expressing DARC. IL-8 and RANTES can also lead to the activation of α4β1 integrin in sickle reticulocytes expressing DARC, contributing to the adhesion of these cells to the endothelium.

While SCD is the first disease for which the molecular basis has been identified (128), the pathophysiology of this disorder remains not fully understood despite decades of extensive studies dedicated to decipher these complex mechanisms. While the consequences of the polymerization of abnormal hemoglobin S were originally described to result in RBC deformability impairment and increased fragility, a large number of abnormalities have been described more recently, such as: the consequences of enhanced hemolysis on decreased NO bioactivity/bioavailability, the consequences of hemolysis and other factors on oxidative stress, the activation of inflammation, the release of NETosis products into the blood, the activation of the alternative complement pathway and the production of deleterious extracellular vesicles. All these biological abnormalities modulate and reflect the clinical severity of the patients. But, during the last years, accumulating evidence shows that each of these abnormalities impacts on RBC physiology and biophysical behavior: NO modulates directly the rheology of RBCs, increased oxidative stress may cause damage to the RBC membrane, accumulation of cytokines in the RBCs may further promote their dehydration and increase their adhesiveness to the vascular wall, accumulation of NETs could participate in hetero-cellular aggregation and accumulation of fragments of the alternative complement pathway may fragilize RBCs. Indeed, one may assume that these recent data suggest a new vicious circle in SCD, starting with impaired RBC rheology and increased RBC fragility and ending with further impairment of RBC, which would further worsen the clinical condition of SCD patients (Figure 1). As our understanding of the complex pathophysiological scheme of SCD has clearly improved during the last decade, further studies are warranted to better describe the relationships between the various abnormalities associated with the most frequently encountered genetic disease worldwide.

## Author Contributions

EN, MR, and PC reviewed the literature, wrote the manuscript, and drew the figure. All authors approved the final version of the manuscript.

### Conflict of Interest

The authors declare that the research was conducted in the absence of any commercial or financial relationships that could be construed as a potential conflict of interest.
